# Leptin signaling and circuits in puberty and fertility

**DOI:** 10.1007/s00018-012-1095-1

**Published:** 2012-08-02

**Authors:** Carol F. Elias, Darshana Purohit

**Affiliations:** 1 Division of Hypothalamic Research, Department of Internal Medicine, University of Texas Southwestern Medical Center, 5323 Harry Hines Blvd, Y6-220B, Dallas, TX 75390-9077 USA; 2 Division of Endocrinology and Metabolism, Department of Internal Medicine, University of Texas Southwestern Medical Center, Dallas, TX USA

**Keywords:** Hypothalamus, Energy balance, HPG axis, Reproduction, Neuroendocrine regulation

## Abstract

Leptin is an adipocyte-derived hormone involved in a myriad of physiological process, including the control of energy balance and several neuroendocrine axes. Leptin-deficient mice and humans are obese, diabetic, and display a series of neuroendocrine and autonomic abnormalities. These individuals are infertile due to a lack of appropriate pubertal development and inadequate synthesis and secretion of gonadotropins and gonadal steroids. Leptin receptors are expressed in many organs and tissues, including those related to the control of reproductive physiology (e.g., the hypothalamus, pituitary gland, and gonads). In the last decade, it has become clear that leptin receptors located in the brain are major players in most leptin actions, including reproduction. Moreover, the recent development of molecular techniques for brain mapping and the use of genetically modified mouse models have generated crucial new findings for understanding leptin physiology and the metabolic influences on reproductive health. In the present review, we will highlight the new advances in the field, discuss the apparent contradictions, and underline the relevance of this complex physiological system to human health. We will focus our review on the hypothalamic circuitry and potential signaling pathways relevant to leptin’s effects in reproductive control, which have been identified with the use of cutting-edge technologies of molecular mapping and conditional knockouts.

## Introduction

Leptin is a hormone primarily synthesized and secreted by the white adipose tissue [362]. It is encoded by the *Lep/LEP* gene (previously named *ob* gene, for obese) and circulates in the plasma in free and bound forms. Leptin receptors, encoded by *Lepr/LEPR* gene are found in six isoforms expressed in a variety of organs and cell types [4, 75, 124, 238, 243, 318, 361]. In the last 15 years or so, leptin’s crucial role in multiple endocrine, metabolic and autonomic functions has been unraveled. In the present review, we will highlight the role of leptin in the control of the reproductive neuroendocrine axis. Innumerous studies have described a critical role for leptin in gonadal function, uterine physiology, pregnancy, and implantation, which will not be explored in this review. For discussions on these issues, we recommend consulting the following articles [40, 51, 130, 150, 166, 207, 271, 321]. Herein, we will give special attention to recent advances in the identification of key hypothalamic sites and signaling pathways relevant for leptin’s action in reproductive control.

## Leptin action as a signal of energy sufficiency

Under physiologic conditions, circulating levels of leptin are highly correlated with stored adipocyte mass [83, 215, 362]. Leptin levels fall quickly during starvation, in parallel with the stereotypical response of various neuroendocrine systems and, therefore, fluctuations in the levels of leptin are viewed as a key metabolic cue for the neuroendocrine adaptations that occur during negative energy balance [6, 61, 68, 124]. Falling leptin levels signal energy insufficiency, inducing counter-regulatory responses to preserve or accumulate energy. Increases in appetite and motivation for food search, decreases in thermogenesis and locomotor activity, inhibition of the thyroid axis and activation of the hypothalamic–pituitary–adrenal axis are examples of critical adaptive responses [2]. Additionally, likely due to the high energetic costs of reproductive processes such as pregnancy and lactation, states of negative energy balance rapidly inhibit the reproductive function. Fasting rodents and primates exhibit decreases in sex steroids, pulsatile luteinizing hormone (LH) secretion and fertility [52–54, 215, 221, 258, 339]. Studies from several laboratories, using different species and paradigms, have demonstrated that leptin administration blunts the fasting-induced suppression of LH secretion, restores female cyclicity and improves fertility [5, 140]. In mice, 48 h of food deprivation led to decreased LH levels and longer estrous cycles. Chronic leptin administration sustained elevated LH secretion and precluded estrus delay [5]. Likewise, chronic administration of leptin prevented the reduction of LH pulse frequency in fasted female rats, and maintenance of steady physiologic levels of leptin allowed a preovulatory LH surge in fasted rats [245, 337] (but see also [324]). Acute leptin administration to food-deprived male or female rats induced a rapid increase in LH pulse frequency and amplitude [98, 140], and intracerebroventricular administration of leptin antiserum disrupted cyclicity and LH secretion in fed rats [60].

Similar effects of leptin are less clear in non-human primates as only very high doses of leptin can sustain LH levels in fasted monkeys [122, 191, 192, 220]. Moreover, seasonal breeders (e.g., sheep and hamsters) appear to have evolved divergent strategies to assure reproductive success, favoring photoperiodic cues over metabolic signals. For discussion of species differences and contradictions please see [262, 288]. However, as a valuable proof-of-principle, leptin treatment increased pulse frequency and mean levels of LH, estradiol, ovarian volume and number of dominant follicles in women with hypothalamic amenorrhea resulting from extreme exercise and weight loss [68, 206, 234, 335, 341]. Therefore, despite species differences and controversies in the field, it is now well-accepted that leptin is a key metabolic cue that signals energy sufficiency to control adequacy and timing of reproductive function.

Recently, a series of compounds with the ability of blocking leptin action were produced, including leptin antagonists and antibodies against the leptin binding domain of LepR [120, 254, 303]. With these tools, temporary/reversible blockade of leptin action became feasible and, therefore, the use of these compounds is expected to contribute to our understanding of leptin physiology. For example, treatment of rats with leptin antagonist during early postnatal life impaired the appropriate development of several organs, including ovaries with marked decrease of primordial follicles [12]. This study highlights a previously unrecognized role of leptin in postnatal maturation of reproductive organs.

## Effects of leptin deficiency in the reproductive neuroendocrine axis

The genetic mutation causing leptin deficiency was first identified in C57BL/6 mice (named *ob/ob*, for obese) at Jackson Laboratories [165]. The mutation was later defined as autosomal recessive, and homozygotes are morbidly obese due to hyperphagia and decreased energy expenditure and display multiple neuroendocrine abnormalities [79, 362]. Leptin-deficient mice of both sexes are infertile, but some degree of reproductive capability has been described in young *ob/ob* males [194]. At prepubertal stages, the reproductive organs of *ob/ob* mice are indistinguishable from wild-type, but neither males nor females attain sexual maturation. Proper sexual development and fertility is only achieved if leptin is provided [21, 70, 239]. The infertility phenotype observed in leptin-deficient mice is dependent on genetic background, as obese *ob/ob* mice crossed onto a BALB/cJ strain have improved fertility. The effect of the so called, but yet unidentified, genetic modifiers appears to be sexually dimorphic. Males from the F2 generation are partially fertile, whereas females only show improved fertility after ten generations of backcrossing of the *ob* mutation onto the BALB/cJ genetic background [266].

The reproductive organs of leptin-deficient male and female mice (in C57BL/6 background) display a series of morphological and biochemical abnormalities. The weights of ventral prostate and testes are decreased while the reported weights of the seminal vesicles are variable [239, 317]. The seminiferous tubules contain fewer sperm and the Leydig cells are reduced in size [239]. The uterus is immature and ovaries have a comparable number of follicles to wild-type mice at initial stages, but no mature follicles or corpora lutea were detected [21]. The uterus and ovaries respond adequately to exogenous estradiol and gonadotropins [103, 193]. When transplanted to wild-type females, ovaries of *ob/ob* mice produce sex steroids and viable oocytes, and gametogenesis may be restored by exogenous administration of gonadotropins [24, 161].

Leptin-deficient male and female mice show reduced pituitary content of luteinizing hormone (LH) and increased pituitary content of follicle stimulating hormone (FSH) [24, 317]. However, circulating levels of LH and FSH are decreased in both male and female *ob/ob* mice. Increased gonadotropin secretion can be induced by castration, although at a lower magnitude compared to the wild-type [193, 317]. Notably, *ob/ob* mice are highly sensitive to the suppressive effects of sex steroids on gonadotropin secretion, consistent with a prepubertal condition of high negative feedback restraint [317]. Gonadotropes of *ob/ob* mice respond adequately to gonadotropin releasing hormone (GnRH) challenges, and *ob/ob* female mice can ovulate and become fertile if levels of gonadotropins and sex steroids are maintained at physiological ranges [161]. Interestingly, compared to the wild-type, higher GnRH content is observed in the hypothalamus of adult *ob/ob* mice [24]. These findings indicate that leptin-deficient mice have adequate development and functioning of gonadotropes and gonads but deficient GnRH secretion at the expected time of puberty onset. Consistent with this concept, in vitro and in vivo studies have determined that leptin acts primarily in the brain by stimulating GnRH secretion [198, 199, 257, 347, 358].

## Leptin signaling deficiency in humans

In humans, leptin signaling deficiency caused by genetic mutations is a rare condition. In general, two forms of congenital leptin signaling deficiency have been identified: mutation in leptin (*LEP*) gene and mutation in leptin receptor (*LEPR*) gene. Both comprise rare causes of monogenic obesity and infertility. Individual heterozygotes for *LEP* or *LEPR* genes mutation undergo puberty and show apparently normal reproductive life. Therefore, only one functional copy of *LEP* or *LEPR* genes is required for the normal progression of sexual maturation and fertility. Twenty-nine cases of loss-of-function of *LEP* gene and 20 cases of mutation impairing LEPR signaling have been reported in the literature [77, 118, 119, 123, 129, 205, 227, 228, 259]. Affected individuals are usually born with normal birth weights but rapidly gain weight in their first few months of life. They exhibit hyperphagia with aggressive behavior if food is denied, and become morbidly obese. Failure of pubertal growth spurt and lack of secondary sexual characteristics is usually observed [117]. However, one affected Turkish female with *LEP* gene mutation began to have menses after a delay of 20 years. In addition, three women with *LEPR* mutations had irregular menses starting in their third to fourth decade of life, and sex hormones were consistent with their age [255]. Recombinant leptin therapy to leptin-deficient subjects has restored the gonatropic axis, induce puberty, correct menstrual irregularities, increase height and reduce relative fat mass [116]. No therapy has been described for subjects with *LEPR* mutation.

The role of leptin deficiency in constitutional delay in growth and puberty (CDGP) has also been investigated. Subjects with CDGP have lower leptin levels compared to normal controls [31, 137]. They are usually underweight for their height exhibiting decreased adipose tissue mass and low circulating levels of leptin and, therefore, the delayed puberty onset has been interpreted as a consequence of low leptin production [107]. A missense variant in *LEP* gene was identified in one individual with CDGP [241]. The sequence variant was also detected in his mother that exhibited similar features of decreased fat mass and delayed sexual maturation. Interestingly, this sequence variant was associated with delayed puberty in the context of decreased body mass index (BMI) rather than obesity. Because of this unexpected outcome, further investigation is necessary to determine if pubertal delay is directly associated with deficient leptin signaling.

## Leptin as a permissive factor for the onset of puberty

Many aspects of the reproductive physiology are energetically demanding (e.g., territoriality for males or pregnancy and lactation for females) and, therefore, the individual nutritional condition is a key factor in the onset of puberty [91, 108, 272, 322]. Seminal studies by Kennedy and Mitra [182] showed that the time of puberty initiation in rats is correlated with body size, not chronological age. Subsequently, a series of epidemiological studies in humans proposed that a critical amount of body fat is required for proper sexual maturation [126, 127]. Following its discovery as an adipocyte-derived hormone in 1994, leptin was readily recognized as the potential link between energy stores (adiposity) and the progression of puberty.

Due to the infertility of the *ob/ob* mice and the complete rescue of fertility following leptin administration, leptin was initially hypothesized to be the key signal for the onset of puberty. However, it soon became clear that leptin is rather a permissive factor that is required, but not sufficient, for normal sexual maturation. In this respect, a critical evaluation of some apparent controversies is necessary. Different laboratories have reported dissimilar results on the ability of exogenous leptin to advance the onset of puberty in rodents [3, 9, 72]. Leptin administration at low concentrations, which are insufficient to alter metabolism, in female mice induced an advance in the onset of puberty [3]. In prepubertal female rats, leptin increased the secretion of gonadotropin and the expression of ovarian steroidogenesis enzymes and advanced puberty [9]. In another study, leptin administration was linked to decreased food consumption and did not advance the age at puberty onset. However, pair-fed mice used as controls in this paradigm showed delayed puberty onset, suggesting that leptin administration prevented the delayed pubertal development caused by the negative energy balance [72, 73]. This finding is in agreement with studies showing that maintenance of adequate leptin levels prevents pubertal delay in conditions of negative energy balance [143]. In this context, leptin also functions as a metabolic signal of energy sufficiency, as previously discussed. Low leptin levels induced by a negative energy balance halt sexual maturation, which can be restored by leptin administration.

An additional argument in favor of a role for leptin in puberty initiation would be the occurrence of changes in leptin levels or signaling during the pubertal transition. While still controversial, nocturnal increases in leptin secretion before puberty have been observed in both rats and primates [244, 314]. Moreover, mice overexpressing leptin (transgenic skinny mice) showed early appearance of vaginal opening followed by uterine and ovarian maturation, suggestive of accelerated activity of the hypothalamus–pituitary–gonads (HPG) axis [360]. The skinny female mice also showed an advance in reproductive senescence, indicating that in addition to the advance in the onset of puberty, hyperleptinemia may promote reproductive failure at early ages. Whether this represents a physiological correlation to the dysfunctional activity of the HPG axis observed in obese patients still needs to be demonstrated. Along the same lines, recent studies have suggested that the hyperleptinemia observed in young obesity or overnutrition may be the cause of reported advances in the age of puberty onset [33, 62, 108, 322]. For more details and discussion, we recommend consulting recently published articles focused on this specific issue [7, 34, 49, 104, 108, 171].

## Leptin signaling and the reproductive function

The LepR is a member of the class 1 cytokine receptor family and is found in six isoforms, all derived from splice variants of the *LEPR/Lepr* gene [75, 200, 318]. Of the six isoforms, only the LepR long form (302 amino acids intracellular domain) contains a Box 3 motif, which enables the activation of the Janus kinase/signal transducer and activator of transcription (JAK/STAT) signaling pathway [36, 243, 354]. Binding of JAK2 to STAT3 leads to its phosphorylation at Tyr1138 and translocation to the nucleus where it functions as a transcription factor [19, 240]. Thus, the long-form of leptin receptor (a.k.a. LepRb) is recognized as the physiologically relevant signaling isoform. Consistent with this concept, loss-of-function mutations of *LEPR/Lepr* long-form in humans and mice (*db/db* mice) cause metabolic and reproductive phenotypes remarkably similar to leptin-deficient subjects [77, 79].

The selective deletion of leptin-induced STAT3 signaling in cells that express LepR (LRb^S1138^ *s/s* mice) recapitulates most of the *db/db* metabolic phenotype, producing hyperphagia, high adiposity and disruption of the melanocortin circuitry in both males and females [23]. However, these mice show improved glucose homeostasis and increased body length compared to *db/db* mice, and while *db/db* mice are infertile, a relatively high proportion (40 %) of LRb^S1138^ *s/s* female mice are fertile [23]. All LRb^S1138^ *s/s* females displayed estrous cyclicity, development of reproductive organs and ovarian signs of ovulation, supporting the concept that hypothalamic control of reproduction by leptin is mediated by a STAT3-independent signaling pathway [23]. Subsequent studies have reported a seemingly contrasting result. Deletion of STAT3 signaling (STAT3^N−/−^) from the brain (Nestin-Cre mouse model) resulted in hyperphagic obesity, and high plasma corticosterone, glucose and insulin levels, similar to *ob/ob* and *db/db* mice [134]. Males and females were infertile with deficient development of the reproductive organs. In the attempt to evaluate the contribution of STAT3 signaling pathways to the acute effects of leptin, another group used stereotaxic injection to deliver a cell-permeable phosphopeptide inhibitor of STAT3 phosphorylation into the mediobasal hypothalamus. Females with acute blockade of STAT3 in the ventromedial hypothalamus did not show leptin-induced LH secretion during fasting, indicating that STAT3 expression in the mediobasal hypothalamus is required for the acute effects of leptin on fasting-induced suppression of LH secretion [46]. In this study, the authors also used genetically modified mouse models to demonstrate the requirement of STAT3 signaling for the acute effects of leptin on food intake and glucose metabolism [46]. Although these mouse studies have generated some controversies concerning the role of leptin-induced phosphorylation of STAT3 in reproductive function, it is important to stress that STAT3 signaling pathways is not unique to leptin response. STAT3 is a common transcription factor recruited by several cytokines including ciliary neurotrophic factor (CNTF) and interleukin 6 (IL6) [38]. Moreover, and of particular relevance to our topic, estrogen appears to also induce phosphorylation of STAT3 in mediobasal hypothalamic neurons [133]. Deletion of STAT3 from all cells in the brain or stereotaxic delivery of STAT3 inhibitor into the mediobasal hypothalamus may block a critical pathway for the control of reproductive physiology unrelated to leptin signaling. Endogenous and selective blockade of leptin-induced STAT3 rendered a mouse model with metabolic features similar to leptin-signaling deficiency but with improved fertility, indicating that the full effect of leptin on reproduction requires the recruitment of a STAT3-independent signaling pathway.

Several studies support the hypothesis that leptin also engages the phosphoinositide 3-kinase (PI3K) signaling pathways to exert its effects [243, 250, 352, 363]. For example, in hypothalamic slices, leptin hyperpolarizes a subset of arcuate nucleus neurons via activation of an ATP sensitive potassium channel, resulting in reduced firing rates [309, 310]. This effect is fully blocked by PI3K inhibitors [250, 345]. Likewise, the leptin-mediated depolarizing effect on proopiomelanocortin (POMC) neurons is dependent on PI3K signaling and administration of PI3K inhibitors blunted the ability of ICV leptin to reduce food intake [156, 250, 363]. In addition, blockade of PI3K signaling precluded the ability of hypothalamic leptin administration to inhibit white adipose tissue lipogenesis [45].

The mechanism by which leptin triggers PI3K activity is thought to be via phosphorylation of insulin receptor substrate-2 (IRS-2) [47, 249]. As the name implies, IRSs are also implicated in insulin signaling [342], but the extent of overlap between leptin and insulin cellular responses in different organs and tissue is still unsettled. IRS-2 is expressed in hypothalamic sites responsive to leptin and related to metabolic control, including the arcuate nucleus and the ventromedial nucleus of the hypothalamus [256]. Global deletion of IRS-2 (IRS-2^−/−^) in mice cause metabolic dysfunction and female infertility [48]. IRS-2^−/−^ female mice failed to cycle and ovulate, had decreased levels of sex hormones and deficient ovarian development. Interestingly, recent studies have shown that mice with inactivation of IRS-2 specifically in LepR neurons are fertile, but whether these mice display regular puberty onset, cyclicity, sex hormone levels in response to metabolic challenges or advanced or delayed reproductive senescence has not been reported [279].

Multiple forms of PI3Ks exist. The class 1a enzymes are heterodimers consisted of one regulatory and one catalytic subunit. There are typically five regulatory subunits, collectively referred to as p85s, and the catalytic subunits are comprised of three variants referred to as p110s [58, 331]. Activation occurs when p85 binds to IRS and localizes the catalytic activity/subunit to the cell membrane, where PI3K catalyzes the phosphorylation of PIP2 to PIP3 that, in turn, recruits and activates downstream targets. The PI3K catalytic subunits p110α and p110β are ubiquitously expressed, and global deletion of either one is embryonically lethal [29, 30, 69, 159]. However, mice carrying a knock-in mutation causing a 50 % loss-of-function of p110α activity are viable, but displayed suppressed IRS-2 signaling and, thereby, decreased responsiveness to insulin and leptin, hyperphagia, glucose intolerance and increased adiposity [125]. Both catalytic subunits are coexpressed in hypothalamic neurons, which express LepR [345]. Selective deletion of these subunits from LepR-expressing neurons precludes leptin-induced changes in cellular activity [8, 345]. However, whether the lack of leptin-induced PI3K signaling results in reproductive deficits has not been reported.

Another signaling pathway potentially recruited by leptin is the mammalian target of rapamycin (mTOR) [87]. The mTOR is a highly conserved serine-threonine kinase downstream of the PI3K–Akt pathway, which is involved in multiple physiologic regulations. Of importance to this review is its critical function in the control of growth and development via the integration of signals from nutrients and circulating factors [160, 196]. The availability of nutrients (amino acid l-leucine) and adequate levels of adenosine-triphosphate (ATP) activate mTOR that, in turn, induces protein synthesis [147, 160, 287]. Therefore, mTOR is recognized as a sensor of energy availability in cellular growth and development. Mutation in the gene encoding *Mtor* in mice is embryonically lethal, whereas increased activity of mTOR induces metabolic dysfunctions including obesity and diabetes [131, 151, 160, 237]. High levels of constitutive mTOR activity down-regulate insulin and growth factor signaling by decreasing insulin receptor substrate (IRS1/2) expression and protein stability [299, 327]. In agreement, the activation of mTOR pathway is elevated in the liver and in the skeletal muscle of insulin-resistant obese rats maintained on a high-fat diet, whereas absence of a downstream mTOR target, the ribosomal S6 kinase (S6K), protects against diet-induced obesity and enhances insulin sensitivity in mice [183, 328]. Following this line, leptin administration to rodents induced phosphorylation of downstream targets of mTOR (S6K1), and blockade of mTOR by rapamycin precluded leptin-induced phosphorylation of S6K1 and the effects of leptin to reduce food intake [87].

A role for mTOR in the neuroendocrine reproductive axis of the female rats was reported [273, 274]. Activation of the mTOR pathway by acute administration of l-leucine increased LH levels in prepubertal mice, whereas l-leucine chronic treatment resulted in no changes in puberty onset. Likewise, l-leucine administration slightly increased LH levels in caloric restricted prepubertal female rats, but did not correct the delayed onset of puberty typical of this experimental model. On the other hand, blockade of mTOR by central administration of rapamycin delayed puberty and blunted leptin’s effects in promoting pubertal development of food-restricted female rats [273, 274]. It will be important to define whether this effect is directly associated with leptin signaling or with a general blockade of a crucial factor of adequate nutrition and energy availability required for reproductive function.

Recent studies have also suggested a role for Crtc1 (cAMP-responsive element-binding protein regulated transcription coactivator-1) linking energy balance and reproduction [10]. Crtc1 is a cytoplasmic coactivator highly expressed in the brain. It translocates to the nucleus following dephosphorylation induced by cAMP or calcium activation [35, 295]. Two independent groups have reported that mice with a loss-of-function mutation in the *Crtc1* gene develop obesity [10, 42]. However, infertility was only observed in one mutant mouse line [10]. Females from the mutant line generated by Alterejos and colleagues, displayed low gonadotropin and sex steroids levels, and lacked development of the reproductive tract. Interestingly, in this study the authors also demonstrated that leptin increases the dephosphorylated (nuclear) form of Crtc1, and thereby regulates the transcription of specific neuropeptides involved in the control of the reproductive physiology (i.e., kisspeptin) [10]. Because of the contrasting findings observed by independent groups using similar techniques and mouse genetic backgrounds, further studies will be necessary to evaluate the contribution of Crtc1 to leptin’s effects on the reproductive neuroendocrine axis.

## Brain circuitry engaged in leptin’s action on reproductive control: redundancies for the benefit of the species

Leptin receptors are expressed in a wide range of organs and tissues [361]. However, the literature now converges to a common view that most of leptin’s actions, including those in the reproductive neuroendocrine axis, are mediated by the long-form LepR expressed in the central nervous system [78, 95]. The initial observations of dense LepR expression in hypothalamic sites and leptin-induced increases in LH pulsatility in a variety of species and both sexes suggested that GnRH neurons are direct targets of circulating leptin [199, 257, 347, 359]. This concept was later reinforced by the observation that immortalized GnRH (GT1) neurons and GnRH-expressing human neuroblasts (FNC-B4) express LepR and that leptin may directly alter GnRH cell activity [217, 236, 313, 361]. However, the expression of the LepR gene in GnRH neurons has been difficult to demonstrate. Recently, with the use of mouse models expressing reporter genes, it has become evident that mouse GnRH neurons express virtually no or very low levels of LepR [100, 208, 269]. The evaluation of the requirement of LepR expression in GnRH neurons for sexual maturation and fertility came with the use of conditional knockout mice. Quennell and colleagues demonstrated that the deletion of LepR from GnRH neurons produces no identifiable reproductive deficits [269]. Males and females show regular pubertal development, are fertile and retain normal fecundity. This study determined that LepR expression in GnRH neurons, if proven, is not required for leptin’s effects on mouse reproductive physiology. Therefore, it seems reasonable to assume that leptin stimulation of GnRH secretion is primarily mediated by a relay station via interneurons that innervate GnRH cells. The search for these specific neuronal populations has become a critical issue to the field.

LepR is highly expressed in numerous brainstem and hypothalamic sites [59, 113, 121, 231, 232, 242, 294]. In the brainstem, the nucleus of the solitary tract (NTS) might possibly mediate leptin’s effects as it functions as a sensory relay of the internal milieu via vagal afferents or the area postrema [132, 172, 175]. However, a role for the NTS or the brainstem in leptin’s action on reproductive control is unlikely because deletion of LepR selectively from forebrain nuclei (CamKIIα-Cre mouse model) produced male and female mice with reproductive deficits similar to leptin-deficient mice [269]. These findings demonstrate that leptin action in the brainstem is not required for reproductive function. Rather, LepR-expressing neurons located in the forebrain are the main players.

## Stress-induced disruption of the reproductive function: the role of the paraventricular nucleus of the hypothalamus

Food deprivation, severe caloric restriction and negative energy balance are well-described states of nutritional stress [74, 93]. In general, stressors of different origins activate the hypothalamus–pituitary–adrenal (HPA) axis primarily by inducing the secretion of corticotropin releasing hormone (CRH) into the hypophyseal portal system. CRH, in turn, stimulates adrenocorticotropin (ACTH) release from the anterior pituitary gland and subsequent corticosterone production from the adrenal gland [152, 312]. The adenohypophyseal neuroendocrine CRH neurons are located in the medial parvicellular aspect of the paraventricular nucleus of the hypothalamus (PVH), a prominent hypothalamic structure involved in the coordination of a series of neuroendocrine and autonomic responses [282, 284]. Conditions of nutritional stress (i.e., perceived negative energy balance) activate the HPA axis, concomitant with a decrease in leptin and LH secretion [5, 216, 325]. Exogenous leptin infusion during fasting prevents the activation of the HPA axis and the suppression of the reproductive axis [5]. In light of these coordinated responses, several groups have postulated that the negative energy balance-induced decrease in LH secretion might be an inhibitory response triggered directly by the high activity of the HPA axis. The experimental approach selected to test this model was the use of 2-deoxyglucose (2DG)-induced blockade of cellular glucose utilization as a controlled experimental tool that mimics states of starvation. In this paradigm, 2DG increased corticosterone levels, and decreased leptin and LH secretion [246, 325, 338]. Pharmacologic inhibition of CRH signaling in 2DG treated female rats normalized LH levels [325]. However, while leptin treatment prevented the 2DG-induced increase in corticosterone, it had no effect on LH secretion [246]. Two important conclusions can be drawn from these findings: (a) the restraint of LH pulsatility caused by blockade of cellular glucose utilization appears to be mediated by CRH signaling in the brain; and (b) leptin’s induction of LH secretion during negative energy balance is independent of the normalization of HPA axis activity and does not overcome the deleterious effects of lack of glucose availability.

Leptin-deficient *ob/ob* mice also exhibit abnormal stimulation of the HPA axis [106]. Leptin administration to these mice inhibits the activity of the HPA axis by decreasing ACTH and corticosterone levels [11, 148]. However, the direct action of leptin on PVH CRH-expressing neurons is still uncertain and a series of apparent controversies deserves an attentive look. Early studies have proposed that leptin’s effect on food intake may be attained by stimulation—not inhibition—of CRH neurons. For example, intracerebroventricular administration of leptin to fasted rats increased CRH mRNA levels in the PVH [291, 326]. This change was not observed in LepR-deficient Zucker rats, indicating that it is dependent on LepR signaling, not secondary to the stress of the experimental procedure. Leptin also stimulated CRH secretion from hypothalamic explants of rats and adrenalectomized mice [86, 169]. Because it is reasonable to expect that stimulation of neuroendocrine CRH neurons would induce ACTH and corticosterone production, these findings appear contradictory. However, CRH neurons in the PVH do not comprise a homogeneous population. Rather, they consist of neuroendocrine and autonomic components [281, 284]; therefore, data obtained from changes in CRH expression in the PVH as a whole may not be very informative. Moreover, the expression of LepR in the PVH is very low [59, 100, 113, 294] and the colocalization of LepR in PVH CRH neurons has not been clearly demonstrated. It is interesting though that leptin induces a neuronal response (Fos-immunoreactivity) in neurons of the ventral parvicellular subdivision of the PVH (PVHvp) in rats [109]. The PVHvp projects to sympathetic and parasympathetic preganglionic neurons in the brainstem and spinal cord, and a subset of these neurons coexpress CRH [281, 283, 316]. However, whether PVHvp CRH neurons have a critical role in leptin’s physiology is still unsettled. In any event, leptin’s effect on CRH secretion and/or neurotransmission appears to be achieved via indirect and/or independent pathways, and the response of specific neuroendocrine and/or autonomic neuronal populations may be defined by leptin-responsive neuronal inputs. One component of this circuitry is the arcuate nucleus [242, 292], a hypothalamic site, which has been extensively studied in the context of leptin’s physiology.

## Arcuate nucleus: a prime sensor of the internal milieu

The arcuate nucleus (Arc) lies adjacent to the median eminence, a key circumventricular organ located at the base of the hypothalamus [43, 132]. The median eminence contains fenestrated blood vessels that allow diffusion and interchange of bigger molecules (peptides and hormones) between the brain parenchyma and the bloodstream. Leptin can passively penetrate the brain through the circumventricular organs, with the highest levels of labeled-leptin binding observed in the Arc, surrounding the median eminence [20]. In the Arc, LepRb is expressed in a subset of proopiomelanocortin (POMC), neuropeptide Y/agouti related protein (NPY/AgRP) and Kiss1 neurons [22, 71, 306]. POMC and NPY/AgRP neurons mediate a diverse range of leptin’s actions in metabolism [82, 114, 235, 290, 344, 348] and several studies have identified these populations also as key players linking leptin action and reproduction.

The role of the melanocortin system (POMC and AgRP neurons) in the control of the HPG axis has been the focus of contentious debate. This is mainly due to inconsistencies described by independent laboratories using distinct approaches and the incomparable phenotypes of different species, mutant mice and genetically engineered mouse models. Humans with loss-of-function mutations of melanocortin signaling show no reproductive deficits [117, 190, 355], while in ewes, which are seasonal breeders, studies indicate that melanocortins relay leptin’s action in the HPG axis [14, 15]. In rodents, inconsistent findings must be interpreted based on the experimental design and approach. Arc POMC neurons project to the preoptic area and make apparent synaptic contact with GnRH and kisspeptin cells [89, 201]. One important issue to consider is that POMC, a complex preprohormone, generates neuroactive peptides (e.g., β-endorphin/βEnd and α-melanocyte stimulating hormone/αMSH) with seemingly contradictory effects on the reproductive axis and metabolism. αMSH reduces food intake and stimulates lordosis behavior in female rats [6, 82, 141, 349], while βEnd stimulates food consumption and inhibits GnRH/LH secretion [41, 136, 197]. Moreover, in rats, αMSH may stimulate or inhibit LH secretion depending on the internal milieu [66, 293]. Mice with loss-of-function mutations in *Pomc* or melanocortin receptors (*Mc3r* or *Mc4r*) genes develop hyperphagic obesity [50, 81, 162] and subfertility later in life. Likewise, mice with a genetic mutation causing constitutive and ubiquitous expression of the agouti protein (lethal yellow/Ay)—a competitive antagonist of melanocortin receptors—are also obese and display abnormal estrous cycles and subfertility at older ages [142]. Whether the reproductive deficits of these mouse models are secondary to the deleterious effects of their metabolic dysfunction is currently unknown.

AgRP is expressed in a distinct population of Arc neurons and exhibits an antagonistic effect on melanocortin receptors [144, 252]. AgRP administration or pharmacologic blockade of MC4R blunted the LH surge in ovariectomized steroid-primed female rats [286, 336] and suppressed pulsatile release of LH in ovariectomized rhesus monkey [333]. Notably, in male rats, AgRP increased GnRH secretion from mediobasal hypothalamic explants and increased gonadotropins and sex steroids levels when injected into the cerebral ventricles [311].

These observations, although inconclusive, indicated that the melanocortin system might also mediate leptin’s action in the neuroendocrine reproductive axis. Initial findings demonstrated that in female rats starved for 3 days, leptin administration partially restored the LH surge but the effect of leptin was blunted in the presence of MC4R antagonists [336]. However, pharmacological blockade of MC4R did not prevent the ability of leptin to activate the HPG axis in *ob/ob* male mice [157]. Following the same line, deletion of LepR from POMC neurons, AgRP neurons, or both POMC and AgRP neurons caused no reported reproductive deficits in these mice [18, 330]. In this regard, additional information can be extracted from leptin-signaling (STAT3)-deficient mice (LRb^S1138^ *s/s*). In these mice, as in *db/db* and *ob/ob* mice, the expression of hypothalamic POMC mRNA was reduced and AgRP mRNA was increased compared with control mice, demonstrating that STAT3 is required for the regulation of both mRNAs [23]; but while *db/db* and *ob/ob* mice were infertile, *s/s* mice showed improved fertility indicating that normalization of melanocortin levels in the brain is not required for improvement of the reproductive function. It is interesting that deletion of LepR and insulin receptor selectively from POMC cells generated a mouse model with insulin resistance, hyperandrogenism, ovarian abnormalities and late onset of subfertility [154]. However, as noted before, it is not yet clear if the reproductive deficits observed also in this mouse model are caused by disruption of a direct melanocortin action on GnRH secretion or are secondary to their impaired metabolic function. Collectively, these findings indicate that, despite the initial evidence in female rats, leptin action in melanocortin neurons is not required for the development and maturation of the reproductive system in mice.

Studies from two independent laboratories have demonstrated that ablation of AgRP neurons or knockout of one allele of *Mc4r* gene corrected the infertility phenotype of leptin-signaling-deficient mice [167, 351]. These publications clearly demonstrate that, although leptin action in melanocortin neurons is not required for reproductive function, blockade of a likely inhibitory action of AgRP on GnRH secretion is sufficient to improve fertility in leptin-signaling-deficient mouse models.

AgRP neurons coexpress the inhibitory neurotransmitter GABA, and NPY, a potent orexigenic peptide highly implicated in leptin’s physiology [76, 82, 114, 144, 178, 290, 348, 350]. The actions of NPY in reproductive control are highly dependent on several factors including steroid milieu, stage of development and expression of sex steroids receptors [155, 346]. Administration of NPY to ovariectomized steroid-primed rats increases LH secretion [168, 179, 278, 329]. In contrast, in the absence of gonadal steroids or in intact rats, NPY inhibits reproductive function as seen by decreased circulating levels of gonadotropins and sex steroids, decreased weight of the reproductive organs and decreased copulatory behavior in both sexes [76, 230, 260, 270, 353]. Intracerebroventricular administration of NPY antisera or blockade of NPY gene expression by antisense oligonucleotides diminished pulsatile GnRH secretion [176, 353], whereas NPY stimulated the release of GnRH from mediobasal hypothalamic explants and potentiated GnRH-stimulated gonadotropin release from the anterior pituitary gland [90, 177]. These findings may appear contradictory, but it is important to stress that NPY is expressed in several other brain sites outside the Arc, making some of the reports difficult to interpret or reconcile.

Conditions of negative energy balance such as food restriction, excess exercise and lactation, have been associated with elevated levels of hypothalamic NPY expression, and leptin-signaling-deficient obese mice exhibit high levels of Arc NPY [6, 267, 290, 307, 348]. Thus, it is reasonable to believe that high NPY expression in the Arc contributes to the decreased fertility observed in these conditions [13]. To test this hypothesis, a series of mouse models were generated. NPY knockout mice and mice with selective deletion of LepR from NPY/AgRP neurons display normal reproductive function [115, 330], but deletion of *Npy* gene expression in leptin-deficient *ob/ob* mice improve their fertility [115]. One-third of double mutant NPY^−/−^ *ob/ob* male and 20 % of female mice were fertile. Because previous studies have shown that food restriction ameliorates the reproductive capacity of *ob/ob* male mice [194], it will be important to define whether the improved fertility of the NPY^−/−^ *ob/ob* mice is secondary to an improvement of their metabolic profile (e.g., decreased hyperglycemia and reduced adiposity).

The Arc houses one of the most potent regulators of the reproductive physiology, the kisspeptins (products of the *KISS1/Kiss1* gene). A series of excellent reviews focused on the kisspeptin role in reproductive biology have been published in the last few years and may be consulted for further details [80, 251, 263, 320]. Loss-of-function mutations in *KISS1/Kiss1* or the kisspeptin receptor (*GPR54/Gpr54*) genes causes infertility due to lack of pubertal development and hypogonadotropic hypogonadism in humans and mice [92, 96, 195, 297, 323]. A steady increase in hypothalamic *Kiss1* and *Gpr54* gene expression is observed across pubertal development and administration of kisspeptin to juvenile rodents induces vaginal opening, increases LH secretion and induces ovulation [247, 300, 319]. Notably, a subset of Kiss1 neurons in the Arc coexpress LepR but, in this regard, some inconsistencies are apparent. Using dual-labeled in situ hybridization in castrated male mice, Smith and colleagues reported 40 % Kiss1/LepR colocalization [306]. In ovariectomized guinea pig, Qiu and collaborators reported 80 % of Kiss1/LepR colocalization using electrophysiologic recordings and single cell PCR of Arc neurons [265]. In contrast, minimal colocalization has been reported using different lines of female Kiss1 reporter mice and female rats [89, 208, 324]. In female mice engineered to express green fluorescence protein (GFP) in Kiss1 neurons (or Tachykinin 2-expressing neurons), less than 10 % Kiss1/LepR coexpression was reported in intact mice and 15 % colocalization was found in ovariectomized mice [89, 208]. In female rats, no colocalization has been observed [324]. Different techniques or approaches (e.g., dual in situ hybridization, dual immunohistochemistry, single cell PCR, reporter mice), species differences (i.e., mouse, rat and guinea pig) or the existence of an unreported sexual dimorphism in the response of Kiss1 neurons to leptin may account for these discrepancies. However, the percentage of colocalization is not always a good indicative of physiological relevance. For example, only around 30 % of POMC neurons coexpress LepR, but their role in leptin’s physiology is unequivocal [110, 290, 343, 344]. Following this concept, the requirement of leptin action in Kiss1 neurons was assessed with the use of conditional knockout mice (Cre–loxP system) in which LepR was selectively deleted from Kiss1 neurons [101]. Male and female Kiss1-Cre/LepR-floxed mice showed normal pubertal development, sexual maturation and fertility, indicating that direct leptin action in Kiss1 neurons is not required for normal puberty onset and reproduction, in mice. Notably, a recent report demonstrated that female mice with ablation of kisspeptin neurons early in development exhibited no deficits in pubertal development and fertility [226]. While intriguing, this finding suggests that developmental adaptations may be triggered to allow reproduction and species survival. Therefore, it is also possible that systems redundancy and/or developmental adaptations may occur in mice with lack of leptin signaling in kisspeptin neurons. Additional studies will be necessary to test this hypothesis.

Several groups have reported a decrease in Kiss1 mRNA expression and kisspeptin production in conditions of low leptin levels (e.g., *ob/ob* mice or wild-types in negative energy balance) [63, 174, 212, 268, 306]. Moreover, streptozotocin-injected diabetic male rats displayed decreased levels of hypothalamic Kiss1 mRNA concomitant with low circulating levels of leptin, insulin, LH and androgens [64]. Administration of leptin, but not insulin, increased hypothalamic Kiss1 gene expression, and levels of LH and androgens. Collectively, these studies indicate that leptin administration may restore (or increase) Kiss1 mRNA expression in rodents in negative energy balance. Whether this effect is achieved via direct leptin action in Kiss1 neurons or by the stimulation of leptin-responsive neurons that project to kisspeptin, still needs to be determined.

The existence of a complex interplay among Kiss1, POMC and NPY/AgRP neurons in the Arc has been reported [15, 16, 128]. For example, the melanocortin system may influence the reproductive physiology of ewes via actions in kisspeptin-expressing cells [15]. In mice, subpopulations of kisspeptin neurons in the preoptic area and Arc coexpress MC4R and, therefore, are also apt to respond to the melanocortin system [89]. As an alternative pathway, leptin may influence Kiss1 expression via projections from the ventral premammillary nucleus, a brain site recently shown to play a fundamental role in leptin’s action on reproductive control [98, 101, 203, 208].

## Ventral premammillary nucleus: the emergence of a forgotten pathway

Initial evidence for a role of ventral premammillary nucleus (PMV) neurons in reproductive control came from studies in rats focused on the contribution of sex odorants to the activation of the HPG axis. In response to opposite sex odors, males and females from different species exhibit increased circulating levels of gonadotropins and sex steroids [26, 85, 180, 214, 223, 264]. Odors are relayed by the main and accessory olfactory pathways and converge in the medial nucleus of the amygdala which in turn innervates several hypothalamic nuclei related to behavioral adjustments and hormonal secretion, including the PMV [57, 145, 285, 357]. In female rats on proestrus, electrical stimulation of the medial nucleus of the amygdala precipitated LH secretion [25], a response that is blocked in rats with electrolytic lesions of the PMV [27]. Following copulation, PMV neurons appear activated (Fos immunoreactivity), a response that was later shown to be related to odor stimulation [65, 99, 188, 203, 356]. Projections from the PMV are particularly dense in forebrain sites related to the control of the HPG axis, in sexually dimorphic nuclei and in those components of vomeronasal circuitry [55, 275]. In particular, the PMV innervates kisspeptin neurons and GnRH cells in the preoptic area as well as GnRH terminals in the mediobasal hypothalamus/median eminence [37, 101, 203, 208, 275]. In addition, a high-to-moderate density of sex steroids-responsive neurons (estrogen receptors and androgen receptor) is also found in the PMV of rodents [233, 304]. Thus, PMV neurons are likely to integrate and convey sexually relevant signals from external and internal environments to relevant reproductive control sites in the brain.

The PMV houses a high density of neurons expressing LepR. Of these, a high proportion is depolarized (and most likely activated) by leptin [109, 113, 203, 208, 345]. These neurons coexpress the neurotransmitters glutamate and nitric oxide, and directly innervate GnRH cells [101, 102, 203, 208]. Together, these findings indicate that PMV neurons are potentially stimulated by leptin and may directly activate their terminal targets (e.g., kisspeptin and GnRH neurons) via the release of glutamate, a classical excitatory amino acid [39, 219].

The role of PMV neurons as a key site of leptin action in reproductive control has only recently been demonstrated in a series of experiments using conditional knockouts and excitotoxic lesions in rats and mice. Female rats with bilateral lesions of PMV neurons became anestrus during a transient period of 2–3 weeks [98]. After this time, their cyclicity was apparently normalized but, during proestrus, PMV-lesioned rats showed reduced estradiol and LH levels, GnRH mRNA expression and decreased activation of GnRH and AVPV neurons. Leptin administration to fasted PMV-lesioned rats failed to increase LH secretion, indicating that the PMV is a critical site for the stimulatory effects of leptin on fasting-induced suppression of the HPG axis. However, it is important to emphasize that only a subset of PMV neurons coexpress LepR [203] and, therefore, the effects of the excitotoxic lesions might result from disruption of pathways unrelated to leptin’s physiology, but involved in the control of female neuroendocrine reproductive axis. Thus, to assess the role of PMV in leptin’s physiology, a more selective approach was performed using the conditional knockout technique, in which LepR was endogenously re-expressed in PMV neurons of a LepR null reactivable mouse model [84, 101]. Re-expression of LepR selectively in PMV neurons of LepR null female mice induced pubertal development and improved fertility; but, intriguingly, no amelioration of male infertility was observed [101]. These findings demonstrated that leptin action in PMV neurons of female mice is sufficient to induce puberty and improve fertility in the otherwise infertile LepR null mice. They also indicate that leptin’s action in the reproductive function is sexually dimorphic and that alternative brain sites relay leptin’s effects in male’s reproduction. As discussed in previous sections, neurons in the Arc may convey signals from leptin levels to the male reproductive system. The role of LepR in the male PMV neurons still needs to be unraveled.

Due to the expected redundancy of the system, leptin action in PMV neurons may be sufficient but not required for reproduction in female mice. To test this, a more precise approach would be the selective deletion of LepR from PMV neurons using the Cre–loxP system. However, due to the lack of a mouse model in which Cre recombinase is driven by a gene expressed exclusively in PMV neurons, another strategy was used. PMV neurons of *ob/ob* female mice were ablated by the bilateral administration of an excitotoxic agent (NMDA) and leptin was replaced in these mice. In PMV-lesioned *ob/ob* mice, a significant delay in pubertal development was observed indicating that leptin action in the female PMV is required for an adequate maturation of the reproductive system [101]. However, after 35–40 days in the presence of a sexually experienced male, 80 % of the PMV-lesioned mice showed sexual maturation as indicated by the presence of copulatory plugs, suggesting that alternative/redundant pathways (e.g., melanocortin system) were recruited to allow reproductive success. In agreement, deletion of LepR selectively from neurons expressing neuronal nitric oxide synthase (nNOS) delayed pubertal maturation in female mice [202]. LepR and nNOS are colocalized in neurons of several nuclei, including the PMV where colocalization rate is very high [102, 202, 203] reinforcing the relevance of PMV neurons as key site of leptin action in pubertal initiation.

Of note, several studies have suggested that leptin facilitates sexual behavior in female hamsters and rats [135, 334]. The brain pathways involved in this effect by leptin are not known and the involvement of PMV neurons in the control/modulation of sexual behavior in males or females has not been demonstrated. However, it is interesting that a strong interconnection between the PMV and the ventrolateral subdivision of the ventromedial nucleus of the hypothalamus, a key site in female sexual behavior, has been reported [55, 56, 275]. The physiologic relevance of these reciprocal connections awaits further investigation.

## From satiety center to behavioral gate: the many faces of the ventromedial nucleus of the hypothalamus

The ventromedial nucleus of the hypothalamus (VMH) has been the focus of the attention of many laboratories interested in the complex regulation of energy homeostasis since the seminal report by Hetherington and Ranson [153]. This study showed that bilateral electrolytic lesions centered in the VMH induce hyperphagic obesity, characterized by a remarkable increase in the adipose tissue mass. Subsequently, it was suggested that the lesions may have disrupted the internal connectivity of the medial hypothalamic nuclei and that other hypothalamic sites, including the PVH, may have played a major role in this phenotype [114, 139]. The relevance of the VMH in metabolic regulation was reinstated with the demonstration of dense expression of receptors for metabolic cues (e.g., leptin and ghrelin) and the presence of glucosensing neurons [112, 113, 181, 277, 294, 308, 364]. However, the VMH, like most of the hypothalamic nuclei, is not a homogeneous structure. Rather, it is composed of distinct subdivisions with characteristic projection patterns [56]. The ventrolateral subdivision of the VMH (VMHvl) expresses sex steroids receptors, projects to reproductive control sites and plays a well-defined role in the female sexual behavior [56, 94, 233, 305]. On the other hand, neurons in the dorsomedial subdivision of the VMH (VMHdm) are responsive to metabolic cues (e.g., leptin and insulin) and project to sites related to autonomic and circadian regulation [111, 185, 187]. Therefore, neurons in the VMHdm may influence energy balance by modulating autonomic responses, which include thermogenesis, hepatic glucose production, glucose utilization and secretion of insulin and glucagon [28, 88, 186, 210, 248].

The VMH is virtually the only site in the brain where the steroidogenic factor-1 (SF1) is expressed [163, 296]. SF1 is also found in gonads, adrenal and pituitary gland and, as the name implies, it is a key component of the steroidogenic cascade. Mice with deletion of SF1 gene (*Nr5a1*) show adrenal and gonadal agenesis, disrupted VMH formation and infertility [164, 211, 280, 302]. Notably, deletion of SF1 selectively from the central nervous system (i.e., from the VMH) disrupted VMH neuronal connectivity and impaired female reproductive function [184]. These findings make the VMH a potential candidate to mediate the effects of leptin in reproductive control. Using the SF1-Cre mouse model, two independent groups demonstrated that leptin signaling in VMH neurons is required for body weight homeostasis and adequate control of glycemia and thermogenesis [32, 97], but these mice (both male and female) are fertile. Thus, it was concluded that leptin signaling in the VMH is not required for leptin’s effect in reproductive control. But as noted before, developmental adaptations and the redundancy of the reproductive circuitry should be taken into account. Further studies will be necessary to assess whether mice with leptin signaling only in VMH neurons show improvements in any aspect of their reproductive physiology.

## Relevance for reproductive human health

Our understanding of leptin’s biology has largely grown from experiments in rodents, but a series of studies has also been conducted in humans, demonstrating the unequivocal relevance of leptin for human’s reproductive health. Women have higher leptin levels compared to men [229]. This difference is already apparent at birth; leptin is higher in cord blood of female infants [170, 225, 289]. In girls, a direct relationship between the increase in leptin and gonadal steroid levels has been observed during pubertal development, likely consequent to the increase in body fat. In late puberty, leptin levels are also higher in females [158]. Due to its strong positive correlation with BMI, higher leptin in women has been attributed to a higher adiposity [229]. However, in a cross sectional study of 204 subjects, gender differences in leptin levels persisted even when adjusted for body fat [149, 253]. Moreover, studies demonstrated that women also have higher leptin pulsatility over a 24-h period [204, 229, 289]. Although still undefined, a plausible explanation for the gender difference could be the effects of testosterone to likely decrease leptin levels in men [213].

As discussed previously, leptin levels fall in states of acute energy deprivation, and this physiologic response is seen as an adaptive strategy of diverting energy resources away from processes that are not essential for immediate survival. Extreme exercise (e.g., ballet dancers and gymnasts) and anorexia nervosa are associated with low gonadotropin and leptin levels [105, 224, 340]. Nutritional support to patients with anorexia nervosa improved percent body fat, BMI, leptin and gonadotropin secretion [17]. Likewise, secondary amenorrhea has been associated with decreased leptin levels (<2 mcg/l) [189]. Leptin treatment in women with functional hypothalamic amenorrhea caused by increased exercise and decreased body weight improved gonadotropin pulsatility, endometrial thickness, ovarian volume and ovulation [341]. In addition, leptin treatment in 72-h fasted healthy men prevented the disruption of gonadotropin secretion [67].

Obese individuals are usually in a state of hyperleptinemia and leptin resistance [229, 301]. However, the direct adverse effects of high leptin levels on reproduction in obesity are not well defined. Hyperleptinemia has been shown to interfere with ovulation and inhibit follicular growth in mice ovaries [315]. In humans, studies in vitro showed that higher doses of leptin can interfere with the ability of the dominant follicle to produce estradiol by inhibiting the production of androgen substrate and decreasing the aromatization capacity of granulosa cells [1]. In obese men, fat mass and leptin are inversely correlated with circulating levels of testosterone. Moreover, following human chorionic gonadotropin (hCG) stimulation, obese individuals exhibited higher 17-hydroxyprogesterone (17-OHP)/testosterone ratio compared to control lean subjects. These findings suggest the existence of an enzymatic dysfunction in the conversion of 17-OHP to testosterone in the Leydig cells [166]. Whether this effect is caused by excess leptin needs further investigation.

Controversies regarding the association of leptin with polycystic ovarian syndrome (PCOS) exist. One study found that leptin levels are elevated in women with PCOS [44]. However, the investigators did not adjust their data for BMI or fat mass and therefore the relevance of their findings has been debated. Moreover, subsequent studies were not able to reproduce this initial data, refuting the concept that changes in leptin levels might play a role in the etiology of PCOS [138, 222, 261, 276, 298].

Lipodystrophy is associated with low leptin levels. In particular, leptin is marked reduced in individuals with congenital generalized lipodystrophy (CGL) and acquired generalized lipodystrophy (AGL), compared to familial partial lipodystrophy (FPL) and acquired partial lipodystrophy (APL) [146]. The prevalence of infertility and PCOS is higher in patients with lipodystrophy compared to the general population [173, 332]. Leptin therapy improved insulin sensitivity, decreased testosterone levels, increased sex hormone binding globulin (SHBG) and reversed the features of PCOS [209]. Recently, an interesting case has been reported of a young woman with CGL, undetectable leptin levels and primary amenorrhea who after treatment with leptin underwent menarche and adequate sexual maturation. Leptin therapy improved her metabolic complications and reproductive dysfunctions. The patient eventually became pregnant and was able to delivery at 37 weeks of gestation with minimal complications [218].

## Conclusion

In recent years, the use of genetic modified mouse models and the advance of molecular mapping of brain circuitry have generated a considerable amount of information regarding leptin’s action in the reproductive neuroendocrine axis. Using these techniques we have learned that leptin activates glutamatergic PMV neurons and inhibits GABAergic AgRP Arc neurons. Both populations, in turn, impinge on GnRH and/or kisspeptin neurons, potentially modulating the reproductive neuroendocrine axis. The integration of these opposite signals is crucial to the control of timely secretion of GnRH during pubertal maturation and in states of energetic challenges. Importantly, lack of leptin action in one of these neuronal groups does not preclude sexual maturation, indicating that one neuronal population can compensate the effects of the other. These redundancies and systems plasticity are fundamental features in reproductive biology, allowing procreation and species survival. In addition, the identification of alternative signaling pathways recruited by leptin will allow a better understanding of the mechanisms by which leptin acts as a permissive factor in pubertal maturation and reproduction.
